# A Single Bout of High Heels Dancing Causes an Increase in Circulating Markers of Muscle Tissue Degradation and MMP-3 in Young Healthy Women

**DOI:** 10.1155/2023/8852889

**Published:** 2023-06-01

**Authors:** Jamila Silva, Leandro Borges, Eleine Weimann, Beatriz Belmiro Dias, Flavio Gomes Faria, Beatriz Ferreira Salgado, Paulo de Freitas, Tania Cristina Pithon-Curi, Elaine Hatanaka

**Affiliations:** Programa de Pós-Graduação Interdisciplinar em Ciências da Saúde, Instituto de Ciências da Atividade Física e Esportes (ICAFE), Universidade Cruzeiro do Sul, São Paulo, Brazil

## Abstract

Prolonged wearing of high heels can cause chronic injury and inflammation. Herein, we investigated the presence of muscle injury, inflammation, and neutrophil function in young women after a single bout of stiletto dance class. Sixteen volunteers (23.4 ± 3.8 years; 61.7 ± 8.1 kg; 23.4 ± 2.3 kg/m^2^; and 27.2 ± 3.8% body fat) participated in the study. The plasma biomarkers matrix metalloproteinase 3 (MMP-3), muscle damage (myoglobin (Mb), total creatine kinase (CK), and lactate dehydrogenase (LDH)), and inflammation (interleukin 8 (IL-8), tumour necrosis factor-alpha (TNF-*α*), interleukin (IL]-1*β*, and IL-6) were quantified before and immediately after a single stiletto class (60 min) of moderate intensity. After class, our data showed that the plasma concentration of MMP-3, Mb, and CK increased by 56% (*p* = 0.04; *d* = 0.8), 113% (*p* = 0.007; *d* = 1.1), and 21% (*p* < 0.001; *d* = 0.4), respectively. Reactive oxygen species produced by neutrophils and the plasma concentration of IL-8, TNF-*α*, IL-1*β*, and IL-6 were not affected under the study conditions. We concluded that a single bout of stiletto dance class caused muscle damage but did not alter the plasma concentration of proinflammatory cytokines. These findings are crucial in preventing the progress of chronic injuries that are often noted in dancers with synovitis and arthritis.

## 1. Introduction

Dancing combines artistic ability and physical fitness. While some recreational dance modalities can improve physiological and immunological capabilities [1, 2], competitive dances usually promote a higher risk for musculoskeletal injuries and inflammation due to excessive movement repetitions and nonanatomical positions [3, 4]. Additionally, inadequate shoes can generate muscle damage, joint impairment, and chronic inflammation [5], which are the events that are the basis for the development of degenerative diseases. Barnish and Barnish [6] developed a systematic review from an epidemiological perspective to investigate the effect of stiletto shoes on musculoskeletal injuries in human participants without prior musculoskeletal conditions. They found that high-heeled shoes were shown to be associated with hallux valgus, musculoskeletal pain, and first-party injury. Moreover, Luximon et al. [7] also found that the use of high-heeled shoes elevates the risk of straining and falling, and these shoes may also cause ankle sprain or ligament ruptures due to fall and bone fractures or permanent damage. In this context, stiletto dance, a dance style performed with high heels that combines movements from urban dances and jazz, imposes joint effort in the execution of the movement from the ankles to the knees, replacing the neuromechanical actions of natural protection [8]. Therefore, the high-heeled shoe affects the biomechanical landing characteristics, leading to the hypothesis that the stiletto dance could generate joint damage and inflammation.

After muscle injury as a result of the muscular effort, the breakdown of muscle fibers leads to a serum increase in muscle enzymes, such as myoglobin (Mb), creatine kinase (CK), and lactate dehydrogenase (LDH) [9, 10]. Subsequently, the inflammatory response to cellular injuries is started by the infiltration of leukocytes, such as neutrophils, into the damaged tissue. At inflammatory focus, neutrophils produce inflammatory cytokines and reactive oxygen species (ROS). This production needs to be fast and self-controlled to avoid tissue destruction. If not controlled properly, acute inflammation can generate chronic inflammation, decreased strength, and consequent degenerative joint damage.

Among the potential cartilage biomarkers, members of proteoglycan metabolism, such as matrix metalloproteinase (MMP)-3 and others, are included as promising ones. The literature shows that MMP-3 is in part responsible for the degradation of no collagenous matrix proteins in cartilage in rheumatoid arthritis and osteoarthritis [11]. Besides, structural molecules of the joint, or fragments thereof, are understood to be the most promising biomarkers for use following acute and chronic exercise as well as with joint degradation [12], and MMP-3 is considered an adequate tool to evaluate the intense sport's response [13]. A broader discussion on the mechanisms of change in biomarkers of joint metabolism in response to exercise training in physiologic and pathological conditions has been reviewed elsewhere [14].

Although wearing high heels is an imminent risk for muscle injury and joint damage, there is no study on the physiological and immunological consequences imparted by stiletto dance and the resulting implications on dancers' health. Herein, we aimed to quantify the biomarkers MMP-3, muscular injury (CK, LDH, and Mb), neutrophils activation (ROS), and the levels of cytokines (IL-8, TNF-*α*, IL-1*β*, and IL-6) in the plasma of young women before and after a single bout of stiletto dance class.

## 2. Methods

### 2.1. Subjects

After approval by the Ethics Committee of the local university, sixteen women agreed to participate in the research. All dancers had provided written consent before enrolling in the research, and the study followed the Declaration of Helsinki. Inclusion criteria were nonobese women (BMI 18.5 to 24.9 kg/m^2^) aged between 18 to 35 years. Participants were excluded from the research if they had chronic noncontagious degenerative diseases, used anti-inflammatories medication, or were declared to be smokers or to have acute inflammation on the day of collection. Moreover, all participants reported having no history of previous surgery or major injury to weight-bearing joints. The participants did not perform any exercise for 48 hours before the dance class.

### 2.2. Stiletto Class Protocol

The class consisted of three phases, namely, warm-up, the technical part, and the choreographic sequence. The initial part lasted 10 minutes and consisted of muscle and joint warming. The second part of the class lasted 20 minutes and consisted of walking exercises with high heels and exercises for specific movements for hands and arms, posture, and hips. The final 30 minutes of class were devoted to composing a choreographic sequence. The class was monitored using heart rate (HR) monitors (FT7, PolarSP, Brazil), and the class intensity was defined according to the following HR reserve values (mean ± standard deviation of the mean (SD)): 101 ± 20.9 at the beginning of class, 137 ± 17.2 after 10 minutes, 147 ± 22 after 30 min, and 164 ± 16.4 at the end of class.

### 2.3. Sample Collection

Twenty milliliters of venous blood were collected before and immediately after the stiletto dance class. The blood samples were drawn from one of three main veins at the antecubital fossa (the cephalic, basilica, and median cubitals). In each case, the vein was chosen based on the identification of an optimal site by both visual and tactile exploration. The blood samples were drawn into BD Vacutainer® tubes, which were used for plasma collection and neutrophils separation. After collecting the samples, the blood was centrifuged (400 g, 10 minutes), and the serum and plasma were separated from the cell components. Neutrophils were immediately isolated, and cellular function was assessed.

### 2.4. Determination of Creatine Kinase (CK) and Lactate Dehydrogenase (LDH) Activities

Plasma CK and LDH activities were measured according to the methods established by Zammit and Newsholme [15] no later than 24 hours after the collection of the plasma. For the LDH assay, there was a loss of five samples.

### 2.5. Determination of Cytokines

Plasma cytokines were evaluated by enzyme-linked immunosorbent assay (ELISA). The concentration of IL-8, TNF-*α*, IL-1*β*, and IL-6 was determined according to the manufacturer's instructions (DuoSet Kit; Quantikine, R&D Systems, Minneapolis, MN, USA), and the samples were stored for no longer than 3 months.

### 2.6. Neutrophils Purification

The experiments were performed within 1 h of venipuncture. Human neutrophil (>98%) was isolated from the peripheral blood of human donors under endotoxin-free conditions using Histopaque® (Sigma Chemical Co.) according to the manufacturer's instructions. The purity of the cell preparation was higher than 98%.

### 2.7. Flow Cytometric Measurement of Reactive Oxygen Metabolites Using Hydroethidine

To evaluate ROS release, hydroethidine (1 *μ*M) was added to the neutrophil (2.5 × 10^6^ cells/mL) incubation medium and the cells were treated with PMA (54 ng/mL). ROS release was monitored for 30 minutes. The assays were run in PBS buffer supplemented with CaCl_2_ (1 mM), MgCl_2_ (1.5 mM), and glucose (10 mM) at 37°C in a final volume of 0.3 mL. Fluorescence was measured using the FL3 channel of a FACS Accuri C6 cytometer (Becton Dickinson, CA, USA). Ten thousand events were analyzed per experiment [16].

### 2.8. Statistical Analysis

Data are expressed as mean ± SD of the participants. The characterization of outliers was determined by limiting to two SD, plus or minus, and adjusting the values. After applying the normality test (Shapiro–Wilk test), the statistical analysis consisted of parametric (paired *t*-test) and nonparametric tests (Wilcoxon test) using the software GraphPad Prism (INStat; Graph Pad Software, San Diego, CA, USA). For the comparison between conditions with and without PMA stimulus, the Friedman test was used for nonparametric data. Significance was accepted at *p* < 0.05, and the effect size was calculated and interpreted using Cohen's *d*.

## 3. Results

The participants were characterized by (mean ± SD) weight 61.7 ± 8.1 kg, height 1.6 ± 0.0 m, age 23.4 ± 3.8 years, body mass index (BMI) 23.4 ± 2.3 kg/m^2^, body fat 27.2 ± 3.8% (measured by the tetrapolar bioimpedance device: Biodynamics Corporation, 310, EUA), waist circumference 74.1 ± 4.8 cm, hip circumference 98.4 ± 6.5 cm, and waist-hip ratio 0.8 ± 0.1 cm (Table 1).

**Figure 1:**
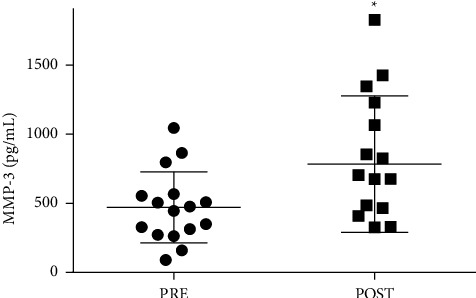
Effect of stiletto dance class on joint injury biomarkers. Plasma concentrations of MMP-3 were determined before and immediately after (I.A.) a dance class. The values are presented as mean ± SD of 16 participants. ^*∗*^*p* < 0.05 for comparison to the pre-class condition.

**Figure 2:**
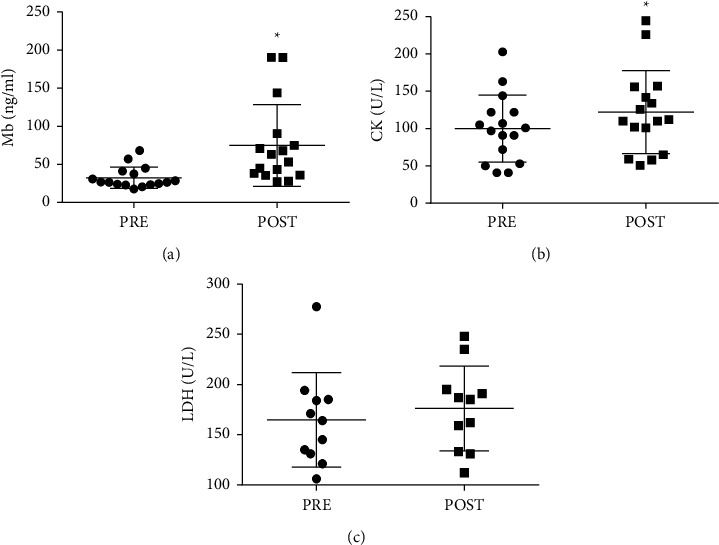
Effect of stiletto dance class on muscle damage. Plasma concentrations of Mb (a) CK (b) and LDH (c) were determined before and I.A. a dance class. The values are presented as mean ± SD of 16 participants for Mb and CK, and 11 participants for LDH. ^*∗*^*p* < 0.05 for comparison to the pre-class condition.

Figure 1 shows the plasma concentration of MMP-3 in the pre and poststiletto class. There was a 56% increase (*p*=0.04; *d* = 0.8) in the levels of MMP-3 after class, and the effect size was considered large.  Figure 2 illustrates the change in plasma protein concentration and activity of muscle enzymes in the pre and postclass moments. The plasma level of Mb (Figure 2(a)) increased by 113% (*p*=0.007; *d* = 1.1), while CK activity (Figure 2(b)) increased by 21% (*p* < 0.001; *d* = 0.3) after class with the effect size considered large and medium, respectively. LDH (Figure 2(c)), under the conditions studied, did not show difference, and the effect size was considered medium (*d* = 0.3).

**Figure 3:**
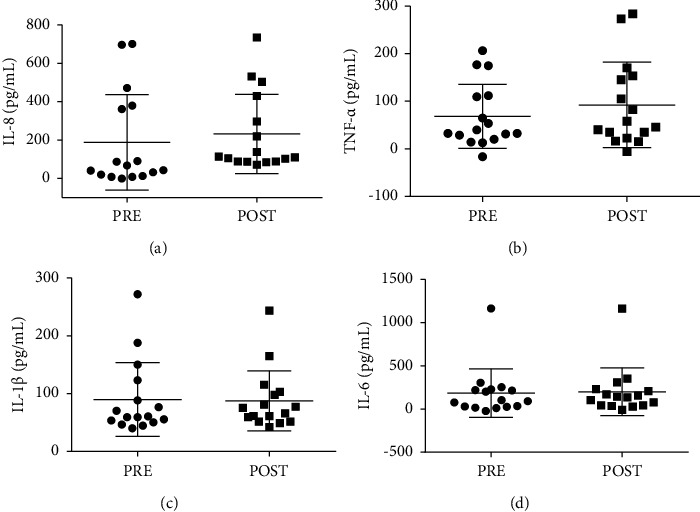
Effect of stiletto dance class on inflammatory biomarkers. Plasma concentrations of IL-8 (a) TNF-*α* (b) IL-1*β* (c) and IL-6 (d) were determined before and I.A. a dance class. The values are presented as mean ± SD of 16 participants.

The plasma levels of proinflammatory cytokines are shown in Figure 3. There was no difference between the moments before and after the class for IL-8, TNF-*α*, IL-1*β*, and IL-6. The effect size was considered small for IL-8 (Figure 3(a)) and TNF-*α* (Figure 3(b)) (*d* = 0.2 and *d* = 0.3, respectively), while for IL-1*β* (Figure 3(c)) and IL-6 (Figure 3(d)), the effect size was insignificant (*d* = 0.04 and *d* = 0.1, respectively).

**Figure 4:**
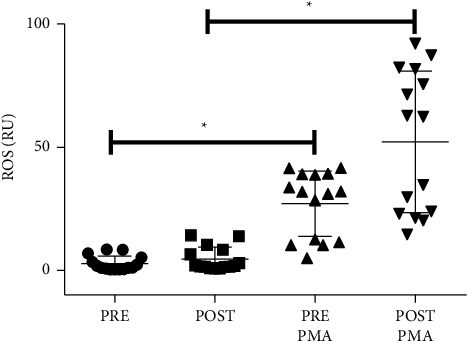
ROS released by neutrophils. Dancers were evaluated before and I.A. a Stiletto dance class. The assessment was performed under basal and PMA-stimulated conditions. The values are presented as the means ± SD of 15 dancers and are presented as relative unit (RU). ^*∗*^*p* < 0.05 for comparison to pre-class condition without PMA; ^#^*p* < 0.05 for comparison to post-class condition without PMA.

Figure 4 presents the production of ROS by neutrophils (with and without PMA stimulation) in the moments before and after a stiletto dance class. As internal quality control of the assay, the difference between conditions was confirmed (with vs. without PMA stimulus) (*p* < 0.05). However, there was no difference between the moments' pre and postclass, and the effect size was considered medium (*d* = 0.4).

## 4. Discussion

Walking in high heels can be challenging with the usual cause of venous complaints, such as fatigue, pain, and heavy-feeling legs, yet they remain a part of many women's attire [17]. The main findings of this research were that stiletto dancing, lasting 60 minutes, increased circulating markers of muscle tissue degradation and MMP-3 after a single class at moderate intensity.

In recent years, CK and Mb serve as biomarkers used to indicate degradation in muscle fibers [18]. After the increase of markers of muscle damage, the symptoms and loss of function during the next hours (respectively days) following these peak values usually involve the conditions of exercise-induced muscle damage: delayed onset of muscle soreness, loss of muscle strength and power, decreased range of motion and systemic increases of myocellular enzymes and proteins, swelling, or an association of these [19]. Commonly, these symptoms last for at least up to 72 h, depending on the extent of disruption of subcellular structures and the volume of muscle-damaging exercise [20]. Our data showed that a single stiletto dancing class increased the plasma level of MMP-3, CK, and Mb. To date, studies have found an increase in these biomarkers in high-intensity exercises [21, 22], which highlights the peculiar character of the use of high heels during moderate-intensity physical exercise.

Given the role of inflammation and synovitis in the progress of degenerative diseases, MMPs may help our understanding of the role of loading on joint health [23]. MMPs in the circulation are thought to regulate the activation of cytokines, growth factors, and angiogenesis, favoring physiological changes to exercise training, and research suggests that the response of catalyzers, such as MMPs, may explain the response of serum joint biomarkers after exercise [14]. Similar to our findings, the MMP-3 level in the blood has been shown to elevate from baseline following a multistage ultramarathon [24]. Curiously, this effect was correlated with the elevation in cartilage oligomeric matrix protein (COMP), implying that MMP-3 may play a function in the degradation of noncollagenous matrix proteins [24]. In this context, an acute bout of high-intensity resistance exercise (6 sets of 10 repetitions at ∼75% 1-RM (one repetition maximum)) has also been demonstrated to acutely elevate MMP-3 in a group of healthy untrained participants [13]; nevertheless, this effect was not noted after an 8-week training program, indicating that training status may impact the response of MMP-3. It is also important to emphasize that outcomes in this field are still preliminary and it is prudent to the development of robust research that investigates the effects of exercise training programs on transcriptional and translational adaptations in the MMP system in skeletal muscle.

Subsequent inflammatory processes are closely associated with muscular damage with a relevant impact on physiological repair mechanisms, as indicated by an elevated invasion of immune cells, such as neutrophils, and usual patterns of pro- and anti-inflammatory cytokines [18]. Moreover, despite the function of matrix metalloproteinases in the degradation of extracellular matrix proteins and tissue remodeling [25], they also influence inflammatory processes through their functional inactivation of cytokines and chemokines [26]. The plasma levels of IL-8, TNF-*α*, IL-1*β*, and IL-6 are associated with the severity of inflammatory processes [27]. In our study, we did not find a difference between these biomarkers before and after the stiletto dance class. The intensity, type, and duration of the exercise can impact the inflammatory response [28], thus, the moderate intensity of the stiletto class (101–164 of the mean HR reserve) may be associated with the permanent concentration of the biomarkers after the proposed class. Besides, the stiletto protocol had a short time duration (60 min), and even if there was a local tissue increase of cytokines, this was not found in the systemic circulation. Therefore, research that targets local tissue concentrations of inflammatory biomarkers associated with the effect of stiletto training could exhibit a promising path in future studies.

Similar to plasma cytokine data, ROS production change can also be related to the intensity of the training. Furthermore, it is important to mention that methodological differences in the assay of ROS, as well as differences in fiber types between the muscles studied, could also be contributory factors to the differences reported in the science sports literature [29]. Studies of our group show that high-intensity exercise in dancers from different styles, such as street dance and classical ballet, can induce a relevant dysfunction of the peripheral blood leukocytes, which is followed by an elevation in proinflammatory mediators [4, 30, 31].

The implications of this research should be interpreted in light of some limitations. First, this is a convenience sample of dancers, and the sample size was small with study participants acting as self-controls, which may limit the generalizability of the findings. Second, the current literature has a scarcity of studies that investigate health parameters in practitioners of the stiletto dance, which makes it hard to compare and discuss the data from the present study with the findings of other dancers. Third, future research could also investigate the effect of a dance class in heels on different biomarkers related to components of cartilage. Regarding the last issue, Bjerre–Bastos et al. recently developed a randomized, cross-over, and exploratory clinical study to assess acute alterations in biochemical markers of bone and cartilage turnover (markers of type I (CTX-I), II (C2M, CTX-II), and VI (C6M) collagen degradation, COMP, and procollagen C-2 (PRO-C2)) in response to moderate intensity and duration (with and without joint impact) in healthy human subjects. They found that running, but not cycling, induced acute changes in biomarkers of bone and cartilage extracellular matrix turnover [32]. Thus, new controlled studies with a larger number of participants are suggested to investigate the effect of stiletto dancers on other joint biomarkers, and the interpretation of these findings should be interpreted with caution.

## 5. Conclusion

In conclusion, an acute bout of stiletto dancing induces an increase in markers of muscle tissue turnover and MMP-3 in young, healthy women. Our data did not indicate changes in markers of inflammation or neutrophil function after the stiletto dancing class. These findings may represent a useful tool to design new studies and strategies to improve the dancer's health.

## Figures and Tables

**Table 1:** Clinical and anthropometric characteristics of the participants at baseline.

	Amateur dancers
Physical and clinical aspects	
Age (years)	23.4 ± 3.8
Weight (kg)	61.7 ± 8.1
Height (m)	1.6 ± 0.0
BMI (kg/m^2^)	23.4 ± 2.3
WC (cm)	74.1 ± 4.8
HC (cm)	98.4 ± 6.5
WHR	0.8 ± 0.1
Systolic BP (mmHg)	117.1 ± 4.3
Diastolic BP (mmHg)	73.9 ± 2.6

*Note*. The values are presented as mean ± SD. Data are represented as mean ± SD of 16 participants. Abbreviations: BMI, body mass index; BP, blood pressure; WC, waist circumference; HC, hip circumference; WHR, waist-hip ratio.

## Data Availability

The data that support the findings of this study are available from the corresponding author upon request.
